# Fish Brain Cell Lines Can Be Infected with Adenoviral Vectors and Support Transgene Expression—An In Vitro Approach

**DOI:** 10.3390/ijms252413357

**Published:** 2024-12-12

**Authors:** Alberto Cuesta, Yulema Valero

**Affiliations:** Immunobiology for Aquaculture Group, Department of Cell Biology and Histology, Faculty of Biology, University of Murcia, 30100 Murcia, Spain

**Keywords:** adenoviral vector, fish, brain, neurotropic virus, European sea bass, gilthead seabream

## Abstract

Host–pathogen interactions and the design of vaccines for aquaculture fish viruses are challenging and call for innovative approaches. This study explores the potential of adenoviral (Ad) vectors Ad5 and chimeric Ad5/40 as gene delivery tools for fish brain cells susceptible to neurotropic viruses. For this purpose, European sea bass (*Dicentrarchus labrax*) DLB-1 and gilthead seabream (*Sparus aurata*) SaB-1 brain cell lines were infected with Ad5 or Ad5/40 vectors expressing GFP, and we evaluated their capacity for infection by fluorescence microscopy and flow cytometry, as well as their antiviral innate immune response by the transcription of gene markers (*irf3* and *mx*). We found that both vectors are able to infect DLB-1 and SaB-1 brain cell lines to similar levels, as demonstrated by fluorescence microscopy and flow cytometry, though the infection efficiency was low. In addition, infection with Ad vectors regulated the transcription of genes related to the interferon-mediated antiviral immune response. Our results indicate that the Ad5/40 vector achieves better infection and consistent cellular distribution. These findings suggest that these vectors may offer targeted gene delivery and local immune responses.

## 1. Introduction

Over recent decades, fish vaccination has become the most effective method for preventing infectious diseases in aquaculture, particularly those caused by viruses [1]. The predominant strategies in both approved and experimental vaccines involve the administration of inactivated or attenuated microorganisms, with or without adjuvants [1]. However, developing effective viral vaccines poses a significant challenge due to the unique physiological characteristics of fishes as poikilothermic specimens and their environmental conditions, complicating the design and generation of fully protective vaccines for aquaculture.

Adenoviral vectors (Ads) are non-integrative modified viruses known for their versatility in delivering foreign genes to cells through the transient expression of the inserted transgene [2]. Beyond being widely used in neuroscience and cancer gene therapy [3,4], Ads have been extensively used in vaccine development for intracellular pathogens, especially in cases where conventional vaccines show limited efficacy, such as the immunodeficiency virus, tuberculosis, and influenza [4,5]. Most commonly, Ad vectors derive from the Ad group C, serotype 5 (Ad5), which infects cells via the coxsackie-Ad receptor (CAR) thorough its immunoglobulin (Ig) domain [6]. Interestingly, a homologous CAR exists in fishes, functioning as a receptor for Ads [7,8,9,10]. In fact, a competitive inhibitor of the CAR reduces the Ad5 infectivity in the fish CAR+ve CHSE-214 (from the Chinook salmon *Oncorhynchus tshawytscha*) cell line but not in the CAR-ve EPC (from the fathead minnow *Pimephales promelas*) cell line, demonstrating the presence and functional implication of such receptors in Ad5 infectivity in fish [10]. Further studies have also demonstrated that Ad5 vectors can, in vitro, infect different types of fish cell lines or primary cells, as well as rainbow trout (*Oncorhynchus mykiss*) muscle or Japanese medaka (*Oryzas latipes*) brain cells after injection [10,11]. In general, these studies evidenced low infectivity of some of the fish cells with the Ad vectors [10,11], suggesting promising applications in fish but limited effects. Indeed, rainbow trout vaccination with Ads expressing infectious hematopoietic necrosis virus (IHNV) and/or infectious pancreatic necrosis virus (IPNV) proteins showed the production of the respective transgenes, induction of antiviral response, antibody production, and protection upon infection [12,13]. Moreover, when injected directly into the telencephalon of zebrafish (*Danio rerio*) embryos, 56% of them showed clear green fluorescence (GFP) expression [7]. This finding suggests that when Ad-vaccines are administered to the fish, they could cross the haemato-cephalic barrier and enter the brain, allowing the fish cells to be used for local immune response. Therefore, this barrier seems not enough to impede the effectiveness of the vaccine when fish become infected.

This study aims to expand our knowledge and potential application of Ad vectors in Mediterranean Sea fish species. Thus, we evaluated herein the infectivity of two Ad vectors in two marine fish brain cell lines, from European sea bass (*Dicentrarchus labrax*) and gilthead seabream (*Sparus aurata*). We have selected two fish brain cell lines since Ads show very good applications in mammalian neurosciences and because they are also selected as vaccination models against fish neurotropic viruses [14,15,16], specifically targeting viruses that affect the brain [17,18]. The application of these vectors in vaccine development could significantly improve protection against viral diseases in aquaculture.

## 2. Results and Discussion

**Figure 1:**
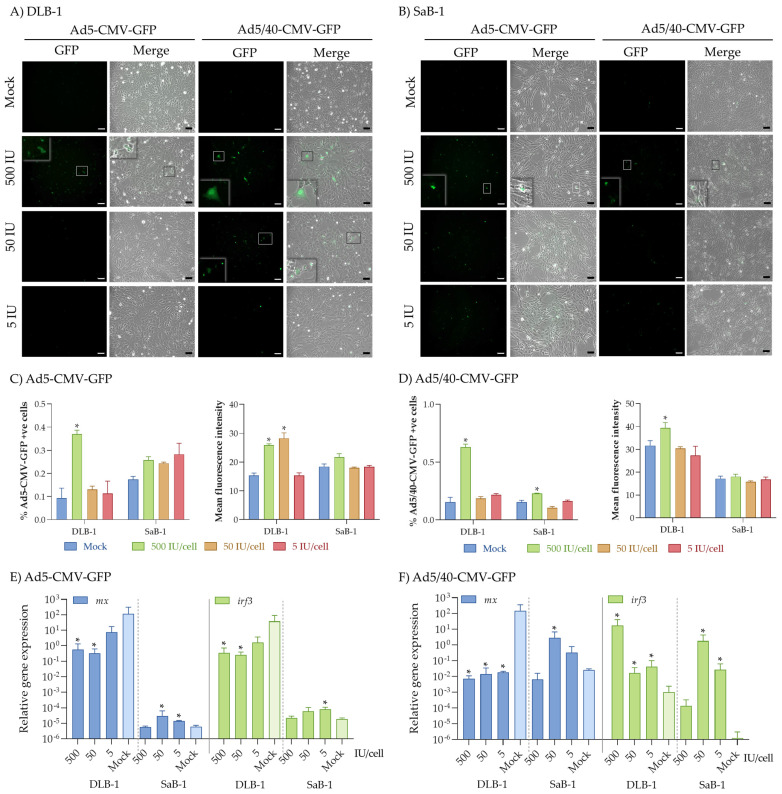
Analysis of the infection efficiency of Ad5-CMV-GFP (Ad5) and Ad5/40-CMV-GFP (Ad5/40) vectors in fish brain cell lines. DLB-1 and SaB-1 cell lines were infected with 500, 50, or 5 IU/cell Ad5 or Ad5/40 at 25 °C for 24 h. Controls consisted of mock-infected cells. Mock-infected cells were treated with culture medium (0 IU/cell). Ad vector expression and GFP localization were observed under a fluorescence microscope (**A**,**B**), while the percentage of GFP+ve cells and mean fluorescence intensity in DLB-1 and SaB-1 cell lines were quantified by flow cytometry (**C**,**D**). Gene expression of *mx* (**E**) and *irf3* (**F**) coding antiviral genes in DLB-1 and SaB-1 cell lines was evaluated by real-time PCR. Bars represent the mean ± standard error of the mean (*n* = 3). Asterisks denote differences with mock-infected cells (*p* < 0.05; Student’s *t*-test). Scale bars: 50 µm.

In this study, we evaluated the tropism of two adenoviral vectors, Ad5-CMV-GFP (Ad5) and a chimeric Ad5/40-CMV-GFP (Ad5/40), in brain cell lines derived from fish species relevant for Mediterranean aquaculture, which are highly susceptible to the neurotropic betanodavirus: DLB-1 from European sea bass and SaB-1 from gilthead seabream [19,20]. The two defective Ads were selected due to their broad tropism [6], which makes them promising biotechnological tools for targeting fish species that express a CAR-homologous receptor. The cell lines used serve as model systems for studying viral infections and immune responses to neurotropic viruses [19,20,21], making them particularly suitable for investigating the potential of adenoviral vectors in fish. Considering the great variability in viral susceptibility and immune responses across different fish species, we chose an in vitro approach. This method facilitated a controlled evaluation of the adenoviral vectors’ performance, enabling a comprehensive evaluation of their potential as versatile biotechnological tools for gene delivery in the fish species under study. Thus, after the in vitro infection, our findings revealed moderate tropism of both vectors towards DLB-1 and SaB-1 cell lines after 24 h of infection (Figure 1A–D), similar to previous observations in common carp (*Cyprinus carpio*) and Nile tilapia (*Oreochromis niloticus*) cell lines [10]. Interestingly, only the highest concentration of Ad vectors, 500 IU/cell, produced a significant infection. For both vectors, fluorescence was distributed in the nucleus and cytoplasm, with the highest detection in DLB-1 cells infected with the Ad5/40 vector (Figure 1A,B and insets). Additionally, we also detected them in cellular granules of both fish cell lines (Figure 1A,C and insets), suggesting potential exocytosis. This process may assist viral release or trigger an immune response, as reported in bovine neuron-like adrenal chromaffin cells [22]. Replication-defective Ads are suggested to alter the exocytotic machinery’s sensitivity or accessibility to calcium, allowing increased exocytosis without a proportional rise in intracellular calcium [22]. Also, a shift from synchronous to asynchronous exocytosis in Ad-infected cells was reported, potentially influencing information transfer in neural cells [22], which deserves further investigation to use Ads as vectors for exogenous expression studies.

**Figure 2:**
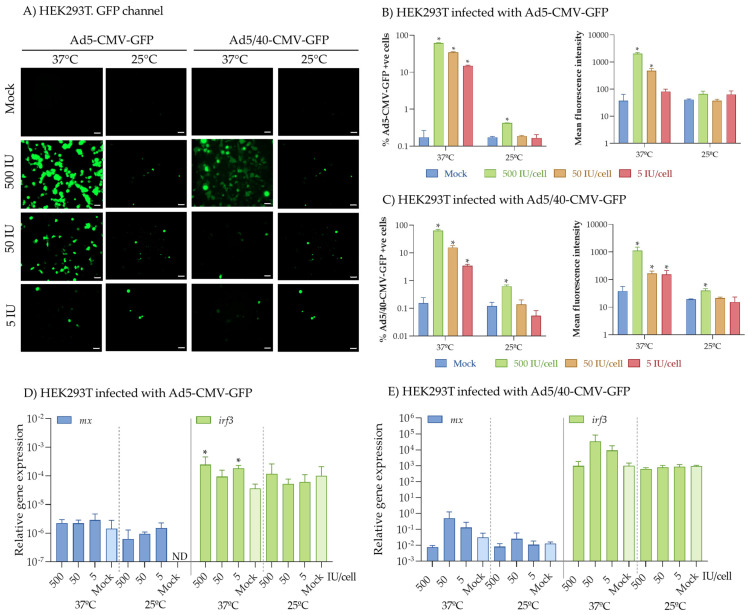
Analysis of the infection efficiency of Ad5-CMV-GFP (Ad5) and Ad5/40-CMV-GFP (Ad5/40) in HEK293T cell line. HEK293T cells were infected with 500, 50, or 5 IU/cell Ad5 or Ad5/40 at 37 °C or 25 °C for 24 h. Mock-infected cells were treated with culture medium (0 IU/cell). Ad vector expression and GFP localization was observed under a fluorescence microscope (**A**), while the percentage of GFP+ve cells and mean fluorescence intensity in DLB-1 and SaB-1 cell lines was quantified by flow cytometry (**B**,**C**). Gene expression of *mx* (**D**) and *irf3* (**E**) coding antiviral genes was evaluated by real-time PCR. Bars represent the mean ± standard error of the mean (*n* = 3). Asterisks denote differences with mock-infected cells (*p* < 0.05; Student’s *t*-test). Scale bars: 50 µm.

The application of flow cytometry also allowed us to quantify the percentage of cells producing the GFP as well as the intensity of the fluorescence (Supplementary Figure S1), parameters that served to quantify the infection and replication potential of the Ad vectors. Interestingly, both Ads demonstrated higher infection efficiency in the DLB-1 cell line compared to SaB-1, as indicated by the percentage of positive cells and mean fluorescence intensity (e.g., fold-change of percentage of positive cells of 3.9 ± 0.2 vs. 1.5 ± 0.1 with Ad5, and 4.1 ± 0.2 vs. 1.5 ± 0.0 with Ad5/40 for DLB-1 vs. SaB-1, respectively; Figure 1C,D). In HEK293T cells, infections with both vectors at 37 °C followed expected patterns, reaching up to 78% of positive cells and a dose-response decrease (Figure 2A–C). Very strikingly, a significant drop in the infection rates at 25 °C was evidenced (Figure 2A–C), with similar levels to those observed in fish cell lines in this and other studies. Unfortunately, DLB-1 and SaB-1 cells cultured at 37 °C did not survive beyond 6 h post-infection since they are not viable at this temperature. The weak expression of GFP in both cell lines suggests limited transduction efficiency of the adenoviral vectors in these systems. Apparently, this could be due to the differences in temperature, as shown with the HEK293T cell line, in which the infection rate decreases when cultured at 25 °C. This fact suggests that infectivity of the European sea bass and gilthead seabream by Ad vectors might be more influenced by the temperature than by the cell type or origin. 

The initial interaction of Ad5-derived vectors with host cells is mediated by CAR, a receptor widely expressed on the surface of many mammalian cells through its two extracellular Ig superfamily domains [6,23]. In fish, the CAR encoding gene has been identified in brain cells, enabling adenoviral entry [7,8,9]. Ad5 chimeras, such as Ad5/40, carry fibers from other Ads to limit their broad tropism [24]. For example, Ad5/40 contains, in its capsid, fibers from Ad5 and only the sort fibers from Ad40 [24]. The addition of the short fibers to the chimeric Ad5/40 gives the vector enteric tropism in mammals, as it is known that the long fibers of Ad40 bind to CAR, while the short fibers do not have a described specific receptor yet [24,25]. Taken together, these findings suggest that both Ad5 and Ad5/40 might use CAR as an entry point to infect DLB-1 and SaB-1 cell lines, as shown in other fish species [9,10]. In fact, a search of European sea bass and gilthead seabream genomes results in the positive and clear identification of CAR gene orthologues. However, the enhanced tropism of Ad5/40 in brain cells could involve differential CAR expression or the implication of additional receptors beyond CAR, indicating the need for further research to explore these alternative pathways.

We also examined the expression of antiviral marker genes (*mx* and *irf3*) 24 h post-infection to assess whether the vectors could trigger antiviral responses. In this case, Mx functions as the final effector of the type-I interferon (IFN) pathway, the most potent antiviral mechanism triggered upon initial contact with a virus in fish, which has been shown to exert direct antiviral activity [26]. Therefore, this is widely recognized as one of the main markers of the IFN antiviral response [26]. On the other hand, IRF3 is a key molecule in the innate immune response, primarily involved in pathways associated with infection responses. These include the modulation of the NF-κB pathway and cytoplasmic DNA-mediated signaling [27,28], but IRF3 also participates in RIG-I-like receptor pathways, leading to the induction of the IFN route during viral infections, such as those caused by the nervous necrosis virus, underscoring its vital role in the antiviral defense [29,30]. Interestingly, infection with either of the Ads caused minimal changes in HEK293T cells (Figure 2D,E). In contrast, while both fish cell lines were infected by Ads with low levels of transgene expression, this expression was sufficient to trigger significant differences in the IFN antiviral response (Figure 1E,F). Specifically, both Ads increased *mx* and *irf3* expression in SaB-1 cells at moderate concentrations. However, in the DLB-1 cell line, Ad5 inhibited the antiviral response, while Ad5/40 significantly down-regulated *mx* and up-regulated *irf3* genes. Certain neurotropic viruses capable of crossing the blood–brain barrier can trigger these large variations in antiviral response between species [30,31]. Indeed, the betanodavirus is a neurotropic virus responsible for viral nervous necrosis across various fish species and can cross the blood–brain barrier within hours [32]. This virus induces differential immune responses, depending on the species, independently of their susceptibility to the infecting strain. For example, markers within the type I interferon pathway, such as the *mx* and *irt3* genes used in this work, were up-regulated in the brain of infected Senegalese sole (*Solea senegalensis*) but down-regulated in European sea bass infected with virulent genotypes [30,33]. Conversely, in the brain of gilthead seabream infected with a genotype to which it is asymptomatically resistant, this pathway was activated [30]. All of these together point to comparable mechanisms of action, and thus initiate similar immune responses. However, further research is necessary to confirm this. Unfortunately, these interspecies disparities were only explored after in vivo betanodavirus infections [30,31,33]. Despite the significant efforts made by the scientific community to develop vaccines for gilthead seabream and European seabass against viruses of aquaculture importance, their effectiveness remains highly limited. Consequently, the development of novel biotechnological tools, such as the adenoviral vectors employed in this study, which can express a transgene of interest in the cells of these species while simultaneously modulating the immune response, presents new opportunities. These advances pave the way for the creation of innovative antiviral treatments and vaccines in a field that remains largely underexplored, reduced to one study in rainbow trout [10], and untapped.

## 3. Materials and Methods

Defective recombinant Ads, Ad5, and chimeric Ad5/40, were obtained from the Viral Vector Production Unit (“Universitat Autònoma de Barcelona”, Barcelona, Spain). DLB-1 and SaB-1 cell lines, derived from the brain of European sea bass and gilthead seabream, respectively, were cultured in Leibovitz’s L-15 medium (ThermoFisher Scientific, Waltham, MA, USA) with 10% fetal bovine serum (FBS; ThermoFisher Scientific) and supplements [2 mM L-glutamine, 100 IU/mL penicillin, 100 mg/mL streptomycin] at 25 °C. HEK293T cells were grown in Dulbecco’s Modified Eagle Medium (DMEM; ThermoFisher Scientific) with 10% FBS and supplements at 37 °C with 5% CO_2_, served as technical controls.

Human and fish cell lines were detached using standard trypsinization, washed, and seeded in 12-well flat-bottom plates (ThermoFisher Scientific) before being infected following a modified protocol [10]. In brief, cells were either mock- or Ad-infected with 500, 50, or 5 IU/cell of each virus in medium supplemented with 2% FBS. Experiments were conducted in triplicate at 25 °C and 37 °C. After 24 h, the cells were washed for further processing. Microscopic analysis was carried out using an Axio Observer 7 inverted microscope (ZEISS, Oberkochen, Germany) to assess the expression of the target transgene (GFP) [25]. Cells were later detached, washed, and analyzed with a FACSCalibur BD flow cytometer (Becton Dickinson; Franklin Lakes, NJ, USA) to ascertain the extent of the viral absorption, as indicated by the percentage of green positive cells and the mean green fluorescence (FL1) intensity for each treatment. In each case, 10,000 events were acquired [25]. Representative data corresponding to cell population and fluorescence histograms are included in the Supplementary Figure S1.

The remaining samples were used for gene expression elsewhere [30]. The RNA was extracted from samples preserved in TRIzol^®^ Reagent (ThermoFisher Scientific, Waltham, MA, USA). After treating 1 µg of RNA with DNAse I, cDNA synthesis was carried out using Superscript IV. Quantitative PCR (qPCR) on a QuantStudioTM 5 Flex instrument (Applied Biosystems, ThermoFisher Scientific, Waltham, MA, USA) was then employed to measure *mx Interferon-induced gtp-binding protein* (*mx*) and *interferon regulatory factor 3* (*irf3*) gene expression in each cell line using SYBR Green for detection. The reaction included a 95 °C hold, followed by 40 cycles of 95 °C for 15 s and 60 °C for one minute. Relative gene expression was calculated as 2^−∆Ct^, with the *elongation factor 1 alpha* (*ef1a*) gene as the internal control, and results were graphed. Primers, custom-designed using the OligoPerfect software tool “https://apps.thermofisher.com/apps/olilgoperfect (accessed on 9 December 2024)” and showed in Table 1, were validated for specificity with melting curve analysis and negative controls.

## 4. Conclusions

In summary, adenoviral vectors Ad5 and Ad5/40 are capable of infecting marine fish brain cell lines DLB-1 and SaB-1, particularly the DLB-1 cell line from European sea bass and the Ad5/40 vector. Notably, Ad5/40 demonstrates better cellular distribution within the cytoplasm, nucleus, and cellular granules, while also initiating an antiviral response similar to that observed in brain infections by neurotropic viruses [30]. In light of these in vitro results, in vivo trials would be necessary to confirm their efficacy in animals. The ability of viral proteins from an adenoviral vaccine to cross the blood–brain barrier, akin to virions, could provide additional benefits by eliciting a targeted local immune response. This innovative vaccination strategy in aquaculture could enhance our understanding of fish immunology and neurovirology, leading to new antiviral approaches and supporting sustainable practices.

## Figures and Tables

**Table 1:** Primer sequences used for gene expression analysis.

Species	Protein Name	Gene Name	Accession Number	Sequence (5′-3′)	
European sea bass	Mx Interferon-induced GTP-binding protein Mx	*mx*	AM228977, HQ237501 AY424961	F	GAAGAAGGGCTACATGATCGTC
				R	CCGTCATTGTAGAGAGTGTGGA
	Interferon regulatory factor 3	*irf3*	CBN81356	F	AGAGGTGAGTGGCAATGGTC
				R	GAGCAGTTTGAAGCCTTTGG
	Elongation factor 1 alpha	*ef1a*	AJ866727	F	CGTTGGCTTCAACATCAAGA
				R	GAAGTTGTCTGCTCCCTTGG
Gilthead seabream	Mx Interferon-induced GTP-binding protein Mx	*mx*	FJ490556, FJ490555, FJ652200	F	AAGAGGAGGACGAGGAGGAG
				R	CATCCCAGATCCTGGTCAGT
	Interferon regulatory factor 3	*irf3*	AM956899	F	TCAGAATGCCCCAAGAGATT
				R	AGAGTCTCCGCCTTCAGATG
	Elongation factor 1 alpha	*ef1a*	AF184170030	F	CTTCAACGCTCAGGTCATCAT
				R	GCACAGCGAAACGACCAAGGGGA
Human	Mx Interferon-induced GTP-binding protein Mx1	*mx1*	NM_001144925	F	CTCCGACACGAGTTCCACAA
				R	GGCGGTTCTGTGGAGGTTAA
	Interferon regulatory factor 3	*irf3*	BC071721	F	AGGGGAGTGATGAGCTACGT
				R	GCTCACTGCCCAGTATGTGT
	Elongation factor 1 alpha	*ef1a*	BC029337	F	GTACTGTTCCTGTTGGCCGA
				R	GCGCTTATTTGGCCTGGATG

## Data Availability

Data are contained in this manuscript.

## Supplementary Materials

The following supporting information can be downloaded at: https://www.mdpi.com/article/10.3390/ijms252413357/s1.
