# Supplementary material for: Fish Brain Cell Lines Can Be Infected with Adenoviral Vectors and Support Transgene Expression—An In Vitro Approach
Source: Int J Mol Sci. 2024 Dec 12;25(24):13357. doi: 10.3390/ijms252413357 (PMC11676386; doi:10.3390/ijms252413357)
Supplement: Supplementary file 1 [file ijms-25-13357-s001.zip › ijms-3339183-supplementary.pptx]

## Slide 1
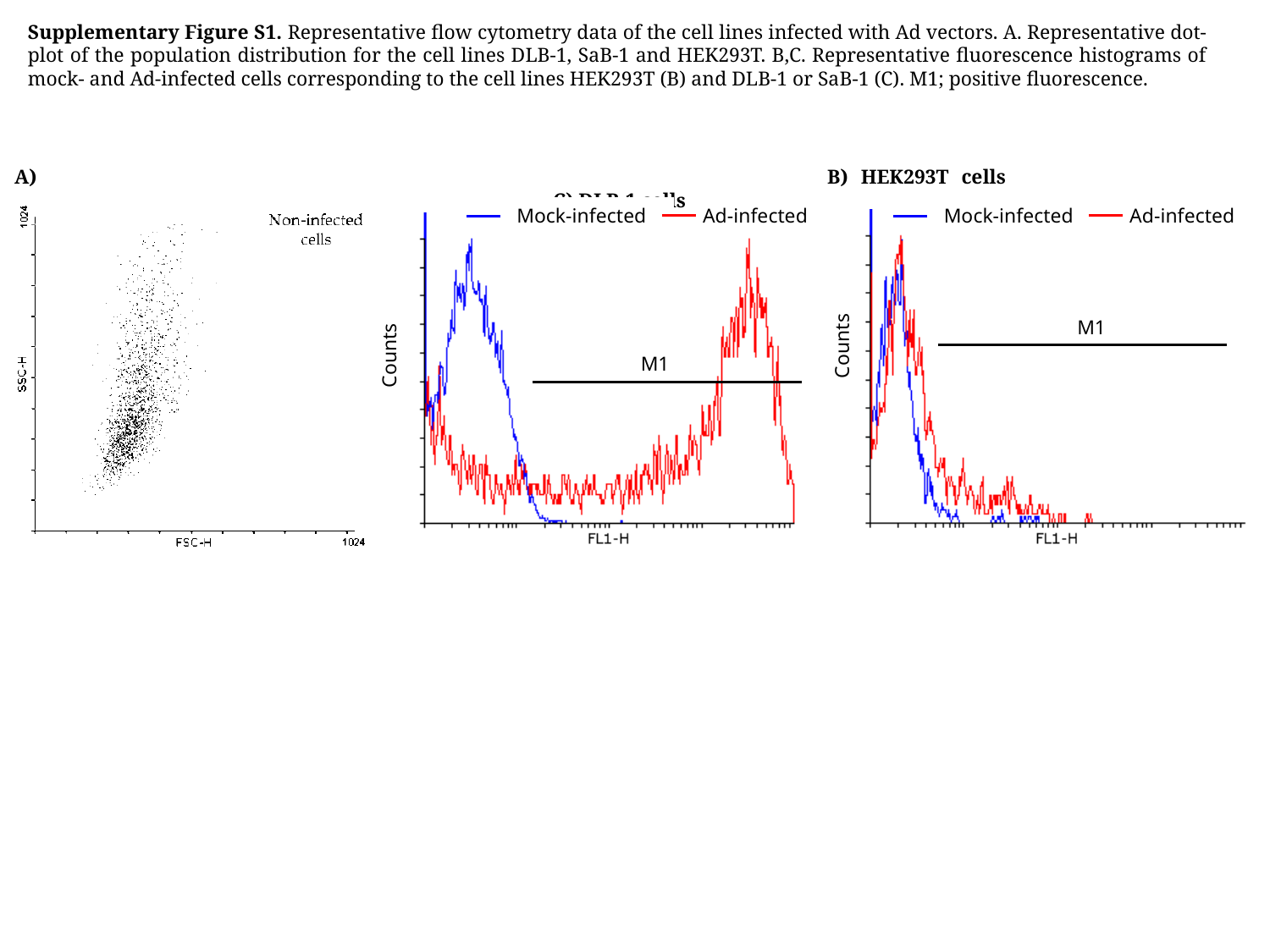

Supplementary Figure S1. Representative flow cytometry data of the cell lines infected with Ad vectors. A. Representative dot-plot of the population distribution for the cell lines DLB-1, SaB-1 and HEK293T. B,C. Representative fluorescence histograms of mock- and Ad-infected cells corresponding to the cell lines HEK293T (B) and DLB-1 or SaB-1 (C). M1; positive fluorescence.
A)						 B) HEK293T cells					 C) DLB-1 cells
Mock-infected
Ad-infected
Mock-infected
Ad-infected
M1
Counts
Counts
Counts
M1
